# Predators, Prey and Habitat Structure: Can Key Conservation Areas and Early Signs of Population Collapse Be Detected in Neotropical Forests?

**DOI:** 10.1371/journal.pone.0165362

**Published:** 2016-11-09

**Authors:** Benoit de Thoisy, Ibrahim Fayad, Luc Clément, Sébastien Barrioz, Eddy Poirier, Valéry Gond

**Affiliations:** 1 Kwata NGO, BP 972, Cayenne, French Guiana; 2 IRSTEA, UMR TETIS, Montpellier, France; 3 CIRAD, Forests and Societies unit, Montpellier, France; Università degli Studi di Napoli Federico II, ITALY

## Abstract

Tropical forests with a low human population and absence of large-scale deforestation provide unique opportunities to study successful conservation strategies, which should be based on adequate monitoring tools. This study explored the conservation status of a large predator, the jaguar, considered an indicator of the maintenance of how well ecological processes are maintained. We implemented an original integrative approach, exploring successive ecosystem status proxies, from habitats and responses to threats of predators and their prey, to canopy structure and forest biomass. Niche modeling allowed identification of more suitable habitats, significantly related to canopy height and forest biomass. Capture/recapture methods showed that jaguar density was higher in habitats identified as more suitable by the niche model. Surveys of ungulates, large rodents and birds also showed higher density where jaguars were more abundant. Although jaguar density does not allow early detection of overall vertebrate community collapse, a decrease in the abundance of large terrestrial birds was noted as good first evidence of disturbance. The most promising tool comes from easily acquired LiDAR data and radar images: a decrease in canopy roughness was closely associated with the disturbance of forests and associated decreasing vertebrate biomass. This mixed approach, focusing on an apex predator, ecological modeling and remote-sensing information, not only helps detect early population declines in large mammals, but is also useful to discuss the relevance of large predators as indicators and the efficiency of conservation measures. It can also be easily extrapolated and adapted in a timely manner, since important open-source data are increasingly available and relevant for large-scale and real-time monitoring of biodiversity.

## Introduction

Despite continuous and increasing local efforts and political initiatives, habitats and species continue to decline worldwide, and projections remain pessimistic [1, 2]. However, long before spectacular deforestation [3], human pressure may result in "empty forests" [4]. Impacts of defaunation are cryptic and pernicious [5]. Population decreases of species with important ecological roles modify ecosystem engineering, seed dispersal and plant recruitment [6–8], as well as community structure [9]. Beyond species loss and their associated functional roles, the loss of ecological interactions may also amplify the consequences of losing ecological services [10].

The pace of global forest habitat loss in South America, most particularly Amazonia, is also on the rise. Amazonian forest ecosystems, like other tropical forests, are also threatened by large-scale defaunation [11]. This forest loss nevertheless remains much lower in the Guiana Shield: Guyana, Suriname, French Guiana and the Brazilian state of Amapá [3,12]. This region is the largest contiguous exposed Precambrian rock in South America, covering more than 2 million km^2^ of northeast Amazonia [13]. This historical absence of large-scale deforestation makes this region the largest repository of tropical forest vegetation on Precambrian terrain in the world [14]. The region is an area of major importance for conservation of several large terrestrial mammals, including peccaries (*Tayassu pecari* and *Pecari tajacu*), Brazilian tapir (*Tapirus terrestris*) [15], giant and neotropical otters (*Pteronura brasiliensis* and *Lontra longicaudis*) [16, 17], and jaguar (*Panthera onca*) [18]. Threats to their survival are nevertheless present and growing in the region. However, much more than agriculture issues and fires, as elsewhere in Amazonia, gold mining is the main threat in the region [19–21], followed by increasing human population growth, unregulated hunting and widespread unsustainable animal harvesting [22,23], all of which threaten the future of vertebrate communities [24].

Carnivores are widely recognized indicators of forest species decline, and they focus attention on broad conservation approaches. Recently, many species have been studied using the latest niche modeling tools to improve knowledge of ecological requirements and conservation opportunities [17,25,26]. Nevertheless, approaches considering the conservation status of both predator and prey species, the relations between their respective status and the prospectives for the future are scarce and difficult to implement. The jaguar (*P*. *onca*) is the largest Neotropical felid in the Americas, considered to be "near threatened" [27], with decreasing populations mainly caused by habitat loss and currently occupying less than 50% of their historical range [18]. The major threats to jaguars in Amazonia are habitat loss, habitat fragmentation and hunting of both jaguars and their prey. The jaguar is not only a relevant umbrella species for wide biodiversity conservation, but can be considered a good indicator of healthy trophic cascades [5,28,29]. The resources required for relevant assessment of population density [30] may nevertheless be beyond the means of most conservation initiatives. Alternatively, the community of large vertebrates, which may be important prey species for large felids, is a good surrogate to determine the human footprint [24], although for many species a relevant assessment of abundance also requires extensive field work [31]. In contrast, recent developments in remote sensing and aerial imaging have provided new approaches to detect habitat disturbance [32–36] and may help identify depleted and degraded areas and/or areas facing a high risk of population collapse with changes on target species communities and ecosystem functions [37].

Therefore, this study explored how predator and prey species as well as habitat structure are interlinked, and more specifically:

1. Jaguar distribution was assessed within the so-called highest-priority French Guiana and Amapá Jaguar Conservation Unit (JCU) [18] where ecological processes are assumed to have remained stable. We used species distribution modeling (SDM) to identify more favorable, appropriate environmental conditions for jaguars at the country scale, expected to be associated with a higher likelihood of long-term persistence [25].
2. Classical camera-trap survey procedures and capture-recapture models [38] were used to assess jaguar density on four sites and to test how predicted environmental conditions are related to field-measured densities.
3. Proxies of jaguar and prey status and key habitats are suggested.
4. Broader discussion is opened on the relevance of felids as proxy for habitat and fauna community status, and how more rapidly acquired field information, associated with open-source data, can be obtained to inform on large vertebrate populations and to provide opportunities for assessment of species conservation status in areas where field constraints are important.

## Methods

### Study area

This study took place in French Guiana, a French administrative unit covering ~84,000 km^2^ located on the northern part of South America, on the Guiana Shield. The Guiana Shield is one of the largest pristine Neotropical rainforest blocks and a floristically distinctive province compared to the Amazonian basin [39]. Eighty percent of French Guiana is covered by upland moist forests implanted on generally well-drained clayic ferralic soils (i.e., Ferralsol and Acrisol; [40]) over altitudes of 0–600 m. Canopy reflectance has defined five main forest types in terms of structure: low dense forests, high forests with regular or disrupted canopy, mixed high and open forests, and *Euterpe* palm forests [41]. Tree species composition varies depending on the relief: very diversified on the all-slope reliefs, the forest is generally dominated by Lecythidaceae on the northern hilly multiconvex reliefs, the Leguminosae-Caesalpinioideae on the central tablelands and Burseraceae on the southern inland plains [42]. The alluvial coastal plain is covered by marsh forests, savannas, transition forests, and herbaceous swamps and is rather narrow on this part of the Guiana shield [43]. Compared to other Neotropical countries, the forest conservation status of eastern Venezuela, Guyana, Suriname, French Guiana and the Brazilian states of Amapá and Pará is still rather favorable. French Guiana benefits from an extensive network of protected areas including five nature reserves, located in patches in the northern half of the country, and a national park in the south, for a total protected area of 23,000 km^2^ (>25% of the country).

### SDM modeling of jaguars: identification of more suitable habitats

Volunteer participation of naturalist networks (environmental NGOs, a public database, scientists) allowed us to record 302 recent sightings (2008–2014) of jaguars on different 0.5 × 0.5-km units. We used a maximum entropy procedure (MaxEnt 3.3.3k, [44]) to estimate the probability distribution of the maximum entropy of each environmental variable across the study area. This analysis has a recognized effectiveness in processing presence-only data and small data sets [45]. To control the likely geographic bias of sighting distribution, the model was forced to use environmental layers restricted to the sampling areas during the learning stage [46]. Predicted areas of occurrence were then projected at the country scale. The model was run using 75% of records for training and the remaining 25% for testing. Five thousand iterations were used with a bootstrap replicate strategy. A 1.0 × 10^−5^ convergence threshold, logistic output format and linear/quadratic regularization values were set. The following predictive environmental data were used to investigate the occurrence of species and to predict more suitable habitats: pluviometry [47]; mean altitude and range of altitude within the grid [48]; vegetation types defined with low spatial resolution remote-sensing data [41]; biogeographic units [49]; and the human footprint representing the distribution and strength of pressures on natural habitats [24]. The addition of environmental data (pluviometry, altitudes, biogeography) and more local idiosyncratic factors (vegetation types, footprint) may help predict broad spatial structures while bionomic variables may tend to present finer grained spatial patterns [50]. The models were interpreted with the AUC test [51]. However, because the AUC test could lead to misinterpretation of model accuracy [52], the null model hypothesis [53] was also used to test the performance of the predictions. We generated 99 random distributions and considered the 95th AUC value as the upper limit of the 95% CI of the AUC. Then, as soon as the AUC value of one species was higher than this 95th ranked AUC, the accuracy of the SDM was significantly higher than expected by chance alone with *p*<0.05.

### Camera trapping: density assessment

**Fig 1:**
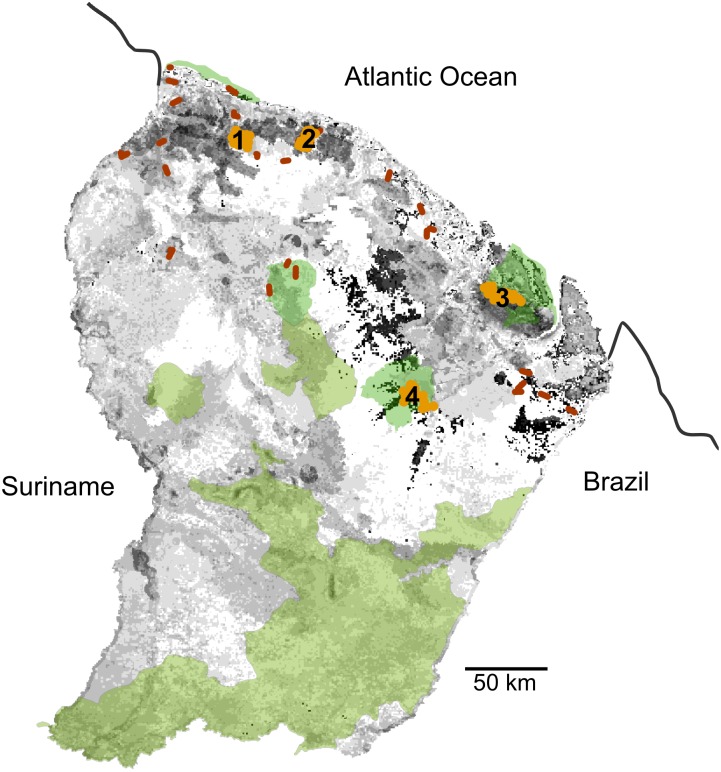
French Guiana study sites, with the protected areas. Protected areas: nature reserves and national parks, in green. Locations of the sites where camera-trap surveys were implemented (1, Montagne de Fer; 2, Counami; 3, Montagne de Kaw; 4, Nouragues) in light brown; locations of the areas where line-transects were implemented in dark brown; and jaguar habitats from less favorable (light grey) to more favorable (dark grey).

Four sites were surveyed in the north and center of French Guiana, in terra-firme highland forests. From west to east, the sites were (1) The Montagne de Fer ("MdeFer", center of the area: 05°20' N, 52°32' W), (2) Counami forest ("Counami", center of the site: 05°18'N, 53°05 W), (3) Montagne de Kaw ("MdeKaw", center area: 04°35' N, 52°20' W), and (4) The Nouragues Nature reserve ("Nouragues", center area: 04°10' N, 52°40' W) (Fig 1). The first two sites are dominated by dense, regular canopy forest [41] and were formerly logged forests. Low-impact logging practices are implemented in these areas, with fewer than five trees extracted per hectare [54] and fully controlled logging activities. The third site was a nearly pristine forest area, partly protected by two nature reserves, Kaw-Roura National Nature Reserve, and Trésor Regional Nature Reserve. The fourth site is a pristine and fully protected area, dominated by dense and regular canopy forest. Sites (1) and (2) did not require specific permission for implementing the studies. Studies on sites (3) and (4) were conducted with the permission formalized by the agreement signed between the National Forest Agency, the NGO AGEP (managers of the Nature Reserve), and the NGO Kwata (in charge of the study).

Surveys were implemented in 2007, 2008, 2009 and 2010 (sites 1, 2, 3 and 4, respectively) during the dry season, from September to December. On each site, 16–18 stations were spaced 2–3 km apart (MdeFer: m = 2.2 ± 0.3; Counami: m = 2.2 ± 0.4, MdeKaw: m = 2.5 ± 0.5, Nouragues: m = 2.4 ± 0.9) with two cameras per station (DeerCam and CamTrakker argentic models for Montagne de Fer, Counami and Montagne de Kaw, and Reconyx 500 for the Nouragues reserve). Traps were in place for 3 months, a reasonable duration to fit with the population closure requirement for CMR models [38]. The surveys included 1,656 station/nights for MdeFer, 1,690 on Counami, 1,530 on MdeKaw, and 1,870 for Nouragues. Data analysis followed classical methodologies, including animal identification based on skin spots, assessment of theoretical population size with capture/recapture methods (CAPTURE, [55]) and assessment of effective study area using recapture distances. A first classical buffer of ½ MMDM (half of the mean maximum distance moved by individuals photographed on separate capture occasions [38]) was used. Half the MMDM may nevertheless result in overestimation of abundance, and a MMDM buffer is recommended [30].

### Abundance of prey species

To investigate the relations between the habitats, the occurrence of the jaguar and the prey community, we explored the abundance of selected jaguar prey species [56,57], including two rodents (*Myoprocta acouchy* and *Dasyprocta leporina*), three Artiodactyla (*Mazama nemoviraga*, *Mazama americana*, and *P*. *tajacu*), and two large frugivorous birds (*Crax alector* and *Psophia crepitans*) on a wide range of sites. Abundance was measured with the line transect method on 30 sites, with a mean area covering 97 ± 2.1 km expected to provide a stabilized assessment of abundance [31] (Fig 1). The 30 sites were distributed in most *terra firme* forest landscape units [40,49], in order to encompass regional particularities related to given forest habitats [58]. Additionnal information on survey sites are provided in S1 Table. The correlations were tested between species abundance (expressed with a kilometric index [KI], i.e., the number of contacts, or individuals in case of gregarious species, per kilometer), the biomass index (KI × mean adult weight, with weights derived from [59] and (i) the predicted occurrence of the jaguar (i.e., the suitability score derived from SDM at the survey site), (ii) the human footprint [24] and (iii) the habitat structure defined with canopy heights and above-ground biomass (AGB) (see below). To assess the area required to target stabilized abundance, we considered the correlation between the asymptotic abundance value (sampling area > 100 km [24]), and abundance assessed with a smaller sampling effort.

### Habitat structure, jaguars and prey

On these 30 sites, four data sources were used to describe habitats: i) maximum canopy height from a full waveform space-borne LiDAR; ii) canopy height as well as roughness and terrain data from the LiDAR waveform length; iii) AGB; and (iv) geology. The LiDAR data were acquired from the Geoscience Laser Altimeter System (GLAS) onboard the ICE, Cloud and land Elevation Satellite (ICESat). The GLAS sensor, which orbited Earth from 2003 to 2009, acquired data using a 1064-nm laser with a nominal footprint on the earth’s surface of approximately 65 m and a distance of 172 m between each footprint. Each waveform contains data on the time variations in the intensity of the energy returned from each laser pulse, thus providing information on the vertical structure of the canopy. Several variables related to vegetation structure can be extracted from the GLAS waveforms [60–63]. However, in this study only the waveform extent, which corresponds to the distance between the signal’s beginning and end, was used. The waveform extent provides information on the combination of vegetation height and the effect of topographic slope [60]. Canopy height estimation resulted from [64], these estimations produced a precision on the canopy height estimation of 3.6 m. Finally, a 3 × 3-pixel spatial standard deviation of terrain data derived from the Shuttle Radar Topography Mission (SRTM) Digital Elevation Model (DEM) were used as a proxy for canopy roughness. AGB estimates stem from a spatial predictive model [65]. This prediction map is based on the inventories of 2507 field plots in undisturbed rainforest (0.4–0.5 ha) distributed over the entire region. The model was developed by kriging-regression in order to include spatial and environmental effects on AGB. The relationship between the niche model, the abundance of prey species, canopy structure, terrain roughness, and AGB was studied using random forests [66]. For the geology, a geological substratum map produced by the French Geological Survey [67] was used. The map was simplified in order to retain only the five largest rock formations: recent sediments, volcanic sedimentary rock, granites, gabbros and gneiss. This simplification was required so that each geology class could be sampled with satisfactory accuracy.

## Results

### Distribution modeling and density for the jaguar

Predicted jaguar occurrence distribution is highly reliable (area under the curve, AUC = 0.832), and the reliability of the null hypothesis that the accuracy of the model was significantly higher than expected by chance was greater than 95% (*p*<0.05). The jackknife test on both test gain and the AUC showed a major contribution of rain and biogeography in the explanation of the distribution. Fig 1 shows the areas where the most favorable conditions were identified with the MaxEnt model. These areas include many habitats in the northern part of the country, in the so-called low joint valleys and complex multiconvex landscapes, and more in the south on low plateaus and mountains (sensu, [42]). On the four sites where density was measured, the number of jaguars identified ranged from six to nine. Depending on the sites and species, CAPTURE identified either Mo (null estimator) or Mh (jackknife estimator) as the best population estimators and calculated eight to ten animals (Table 1). Survey areas calculated on the basis of ½ MMDM (animals captured at more than one camera station, from MdeFer: three (Counami, MdeKaw) to eight individuals (MdeFer); Counami: three individuals; MdeKaw: three individuals; Nouragues: five individuals), were 194, 246, 275 and 229 km^2^ for MdeFer, Counami, MdeKaw and Nouragues, respectively. Associated calculated density values ranged from 2.9 adults/100 km^2^ at MdeKaw to 5.1 adults/100 km^2^ at MdeFer. The survey areas calculated on the basis of MMDM and associated densities ranged from 562 km^2^ and 1.4 adults/100 km (MdeKaw) to 405 km^2^ and 2.5 adults/100 km^2^ (MdeFer). Despite the substantial differences in the number of recorded animals (Table 1), mean densities did not statistically differ between the four sites, and consequently were not influenced by the Human Footprint Index.

**Table 1:** Results of four camera-trapping surveys in French Guiana for jaguars.

	Nb of animals identified / Nb of animals × 100 km^2^*	Calculated population	Mean D max (km)	Surveyed area / density (for 100 km^2^ (1/2 MMDM)	Surveyed area /density for 100 km^2^ (MMDM)
1. Montagne de Fer	9 / 14.0	10 [SE = 3.0]	6.6	194 km^2^ / 5.1 ind.(SE = 3.6–6.7)	405 km^2^ / 2.5 ind. (SE = 1.7–3.2)
2. Counami	6 / 9.0	8 [SE = 2.1]	7.8	246 km^2^ / 3.3 ind. (SE = 2.8–4.1)	530 km^2^ / 1.5 ind. (SE = 1.3–1.9)
3. Montagne de Kaw	6 / 6.7	8 [SE = 1.9]	6.6	275 km^2^ / 2.9 ind. (SE = 2.2–3.6)	562 km^2^ / 1.4 ind. (SE = 1.1–1.8)
4. Nouragues	9 / 10.2	10 [SE = 2.5]	7.1	229 km^2^ / 4.4 ind. (SE = 3.3–5.5)	507 km^2^ / 2.0 ind. (SE = 0.15–2.5)

* as defined by the minimum convex polygon size

### Habitats and prey communities

**Fig 2:**
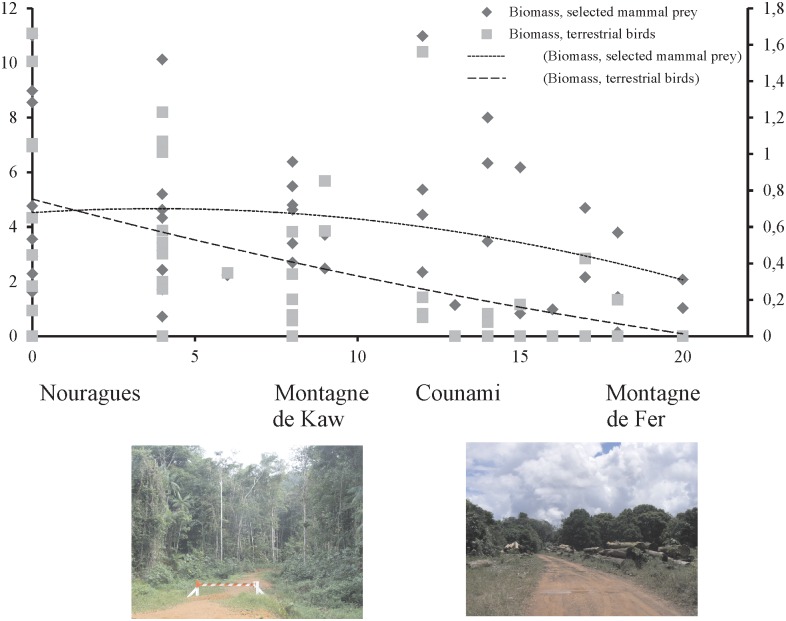
Biomass Index (kg^-1^ × km of transect) of a selected set of terrestrial birds and mammals, according to the Human Footprint Index, and associated correlation curves. The four study sites for jaguar density assessment are given on the index scale, according to their mean value. The two pictures show sites with a 5–10 Index value (left) and a 15–20 Index value (right).

Of the 30 sites, the cumulated abundance of the five target mammal species (*M*. *acouchy*, *D*. *leporina*, *M*. *nemoviraga*, *M*. *americana*, *P*. *tajacu*) and of the two birds (*C*. *alector* and *P*. *crepitans*) were negatively correlated with the human footprint (*p*(uncorr.) = 0.03, r = −0.31 and *p*(uncorr.)<000.1 r = −0.63, respectively). The biomass value of birds was also negatively correlated with the human footprint (r = −0.63, *p* = 0.0002), the biomass of mammals was also negatively correlated to the footprint, although the relation was not significant (r = −0.32, *p* = 0.07) (Fig 2). Although concomitantly measured on four sites only, prey was more abundant where higher densities of jaguars were recorded, but neither biomass nor abundance was correlated with the jaguar’s predicted favorability index.

### Early indicators of population status and collapse?

**Fig 3:**
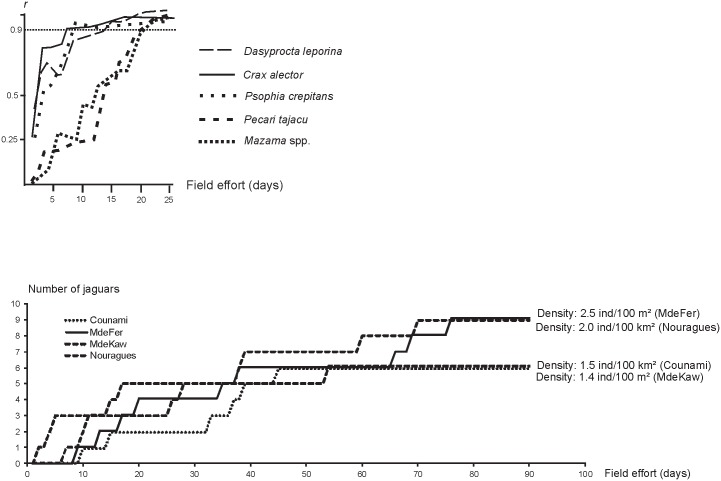
Field surveys required to assess abundance of ungulates and two frugivorous birds, based on 40 surveys (up), and the number of jaguars based on four study areas (down). Top: the r coefficient correlation shows the correlation between the stabilized abundance (sampling effort > 100 km) and abundance assessed with a lesser sampling effort. The correlation is derived from 40 surveys.

The accumulation curves of new jaguars recorded in each survey area stabilized after considerable field work: 50 days (1000 traps/night) on the sites with lower density, and 80 days (1600 traps/night) for the two sites with higher density values (Fig 3). Field work for assessment of high vertebrate abundance shows that a survey longer than 20 days (cumulative distance greater than 80 km of linear transect) is necessary to relevantly assess the abundance of ungulates; the field work required could be shorter for large birds (50–60 km of linear transects, i.e., 8–10 days) (Fig 3).

Canopy roughness was negatively correlated with the human footprint and was higher in more pristine habitats (r = −0.66, *p*(uncorr.)<0.001). The AGB model cannot integrate the effect of human disturbance: remote-sensing data as well as field data on disturbed plots were not included in the analysis [65]. Canopy roughness was correlated with the abundance and biomass of large terrestrial birds (r = 0.73, *p*(uncorr.)<0.001 and r = 0.74, *p*(uncorr.)<0.001, respectively), although relations between AGB and large vertebrate abundance were not significant. Finally, a random forest classification showed that the jaguar distribution on one hand and canopy roughness and geology on the other hand, were significantly correlated (r = 0.61, *p*(uncorr.)<0.01). Adding more information in addition to the canopy structure, such as maximum canopy height from GLAS, GLAS waveform extent, terrain roughness, geology and AGB, the strength of the relation between the niche model and these variables increased (r = 0.71, *p*(uncorr.)<0.01).

## Discussion

In addition to direct habitat loss that can be monitored via direct imaging or with more precise tools (e.g., biomass and carbon measurements [68]), much more cryptic threats such as hunting and its cascading effects comprise the main threat in tropical forests [4], requiring adequate and early indicators. We focused on the region of the Guianas, in northern South America, confronted with a dual challenge: (i) the need to reinforce its recognition as an area of major importance for long-term conservation of forest species and (ii) the need to mitigate increasing human population growth, with its cascades of pressures on forest ecosystems. We developed our approach on predator, prey and habitats, and expect to detect early signs of population collapse, before shifting to empty forests.

### Species distribution modeling and jaguar density

Jaguars were recorded in most of the country, and no large area of local extinction was evidenced. Niche modeling identified some habitats as more favorable, on the northeastern and center relief landscape belts. These favorable regions were previously detected in the wider-scale approach [25] and include "complex multiconvex landscapes," "slopes," "low plateaus" and "mountains" (sensu, [42]). A previous study showed that the *α* diversity of large vertebrates, expected to be prey for predators, was low on those landscapes [58]. But although only a few vertebrate species showed a marked preference for those areas, the red brocket deer (*M*. *americana*), the collared peccary (*P*. *tajacu*) and the red agouti (*D*. *leporina*) were the only species with a clear preference for the complex multiconvex landscapes, low plateaus and mountains of these environments [58]: they may be important resources for the jaguar in these landscapes.

**Fig 4:**
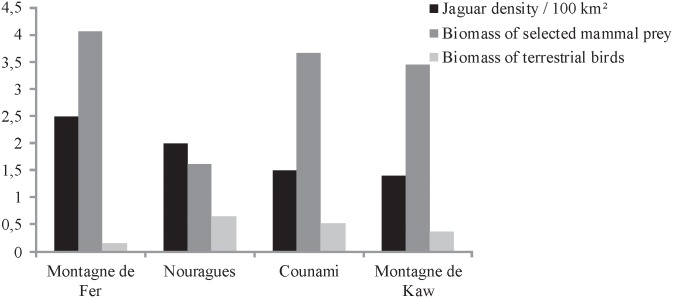
Fauna communities on the four study sites with implementation of both camera-trapping and linear transects: jaguar density, biomass index of mammal prey, biomass index of terrestrial bird prey.

Regarding density, assessments were roughly within the ranges reported in the central and south regions of South America [69] and are expected to reflect healthy populations. Comparisons with other sites should nevertheless be interpreted cautiously, since these data from French Guiana are the first available for the northern Amazon region. At a more local scale, although not significant, differences in felid density were noted between the four sites. The two sites with higher jaguar density were the least disturbed areas: the Nouragues Nature Reserve and the Montagne de Fer, which is a formerly logged area. The network of ancient logging tracks at Montagne de Fer could also have slightly biased the results. In jaguars, the higher tolerance of males to human-modified landscapes, including the attractiveness of tracks [70], increased the capture rate and may have slightly overestimated jaguar density despite the correction by the capture-recapture models. Nevertheless, the rough number of animals detected, weighted by the size of the survey area, remained higher at Nouragues and Montagne de Fer, reinforcing the idea of a large population on the latter site (Fig 4).

The performance of the model, further confirmed by field-measured density, also highlighted the most important areas for the future of populations, directly related to conservation issues. Although the southern areas benefit from National Park protection, the favorable habitats located in the north of the country are less covered by protected areas (Fig 1) and therefore require that particular attention be paid to these areas for future populations.

### Prey and forest structure provide better information on jaguar habitats

Ungulates (*M*. *nemoviraga*, *M*. *americana*, *P*. *tajacu*) and large frugivorous terrestrial birds (*C*. *alector*, *P*. *crepitans*) are expected to be key resources for large felids [71]. When compared to other forest surveys in Amazonia [72–75], the abundance of these species measured in French Guiana confirms the idea of the overall good status of forest ecosystems. However, the general pattern observed of more abundant prey on more suitable habitats for jaguar is not significant. This suggests that the jaguar’s diet may be highly diverse and depend on a wider range of prey species than considered here, including smaller species. Also, we previously evidenced that some opportunistic species, such as agouti, collared peccary and tapirs, may maintain or even take demographic advantage in old logged forests [24,76], explaining the high density observed at Montagne de Fer and helping ensure the future of jaguar populations on those managed forests.

Negative responses of prey abundances to Human Footprint Index (either highly significant in the case of terrestrial birds, or close to significance in the case of terrestrial mammals), suggesting that the mega vertebrate community can persist in selectively logged forests [77], but unmanaged hunting pressure could result in substantial population declines and threatened prey and predators in a cascading effect. Twenty-five percent of the country is above a threshold value, which may indicate that populations of ungulates, large rodents and large terrestrial birds are beginning to collapse [24]. Long before deforestation and forest habitat loss, estimated over the last decade (2000–2012) at less than 20,000 ha, i.e., less than 0.2% of the territory (derived from [3]), overhunting is the main threat for these species. Assessment of hunting sustainability has not been investigated in French Guiana for Artiodactyla, but monitoring of primate hunting has shown unsustainable harvests in many settlements, including in local and traditional communities [23]. Similar results were obtained for tapirs [78]. Given the near absence of hunting management in French Guiana, hunters’ access to large forest areas via logging tracks and hunting pressure exerted by several tens of thousands of gold miners [19,21], the greater threat to jaguar survival could likely be the negative trends of prey abundance due to hunting.

### Regional conservation issues

With wide-ranging species such as large carnivores, together with the range-wide conservation network [79], the identification of refugia and lower-quality habitat are necessary for long-term conservation planning, which may require fine-management-scale units [80]. Human-use areas are also important habitats for connectivity and dispersal of jaguars between core protected areas [81].

Setting priorities for conservation in largely undisturbed regions such as the Guianas may nevertheless differ from those commonly applied to highly disturbed and human-dominated regions [82]. The Amapa–French Guiana Jaguar Conservation Unit of has been listed as the highest priority, with substantial opportunities for large-scale dispersals due to lack of fragmentation with highly conserved Southern forest blocks, and an estimated population size of more than 500 animals [83]. Although this very open and unfragmented population precludes adequate population viability analysis [84], many parts of the Guianas still provide unique opportunities to conserve species with large ecologic and spatial requirements and are expected to ensure its future. The first lesson of this long-term survey of jaguars and their prey is the good status of conservation in French Guiana and likely the entire eastern part of the Guiana shield, with no evidence of widespread jaguar depletion. Governmental management of forests, selective and reduced-impact logging techniques [85] and the network of protected areas have safeguarded a satisfactory status of forest habitats, associated species and trophic cascades. Although the SDM approach identified less favorable habitats (Fig 1), these areas are unfragmented, far from anthropic threats and connect areas of importance. They consequently should not be considered as "ecological traps" (sensu [80]) but as landscape connectivity between preferred areas.

Regarding habitat loss, gold mining activities [15] are responsible for an annual loss of 2,000 hectares of forest habitat, and the recent alarming increase of deforestation due to the demand for gold has been spotlighted [17]. However, long before deforestation, defaunation and empty forests threaten tropical ecosystems. The main concern in the Amapá and French Guiana Jaguar Conservation Unit is overhunting of prey. A decades-long lack of hunting regulation, widespread and cryptic harvests of wild species by goldminers, and demographic expansion of local communities with little access to alternative resources result in silent shifts of rich forests and empty areas. Similar to other Amazonian regions spared by large-scale deforestation [23,86], insufficient management of hunting is likely the most significant threat for ungulates, large birds and the top predators.

### Beyond the Guiana jaguars and canopy: new tools and opportunities for wildlife conservation

Conservation initiatives require adequate proxies to detect early and cryptic population collapses and often suffer from lack of data and limited access to large field inventories. The relevance of the proxies is based on four assumptions: (i) comprehensive sets of input data, (ii) relevant explanatory variables (i.e., environmental descriptors), (iii) good indicator species and (iv) adequate modeling procedures. This raises the question of the target species and the relevance of charismatic species (i.e., species that will draw more attention from nonprofessional recorders) as proxies. Large carnivores and apex predators are widely accepted as indicators and keystone species, and furthermore are often threatened [87,88]. However, the jaguar has recognized opportunistic behavior and diet plasticity with a wide range of prey consumed, including small species [89] that may benefit from forest cover changes [90], for which forest managers may miss early signs of population disturbance and delay detectable responses, are consequently insufficient early indicators of forest and animal community disturbances, at least at the relatively low extent and strength levels observed in the Guianas. We can concur with previous research [91] and assume that large carnivores may not be locally relevant indicators for local and/or early changes in forest animal communities encountering rather low and cryptic threats.

The abundance of some prey species may be a better proxy for the threat to jaguars. However, field work for assessing large vertebrate density and abundance in forest habitats is also important and could delay the ability to detect a population’s collapse. For instance, we previously showed that a cumulative distance greater than 100 km of linear transect could be necessary to relevantly assess the abundance of ungulates [24]: regular monitoring in protected areas and/or in managed forest areas may be limited. The field work required could be shorter for large birds (50–60 km of linear transects, i.e., 8–10 days) (Fig 3). Focusing on these species only, which are also the first to collapse [24], could provide a relevant indicator.

Large sets of open and/or shared data [92,93] allow modeling, extrapolation and assessment of population trends at uncovered resolution and open new avenues for conservation. Increasing enthusiasm for participative science, or "public participation in scientific research," is accepted as a win–win model [94], not only because it results in increased knowledge of biodiversity issues and of the participants’ local environments, but also because engagements in science are expected to change attitudes and environmental behavior [95,96]. In the present case, more than 75% of the jaguar records come from non-scientist recorders, obviously facilitated by the charismatic value of the species. Once such data sets are properly analyzed, they may successfully contribute to biodiversity monitoring. Species distribution models are increasingly used for conservation science [97] and can efficiently contribute to this task, transforming citizen data into identification of areas acting as sources and corridors, and providing original conductance and/or resistance values for landscape connectivity modeling [76].

**Fig 5:**
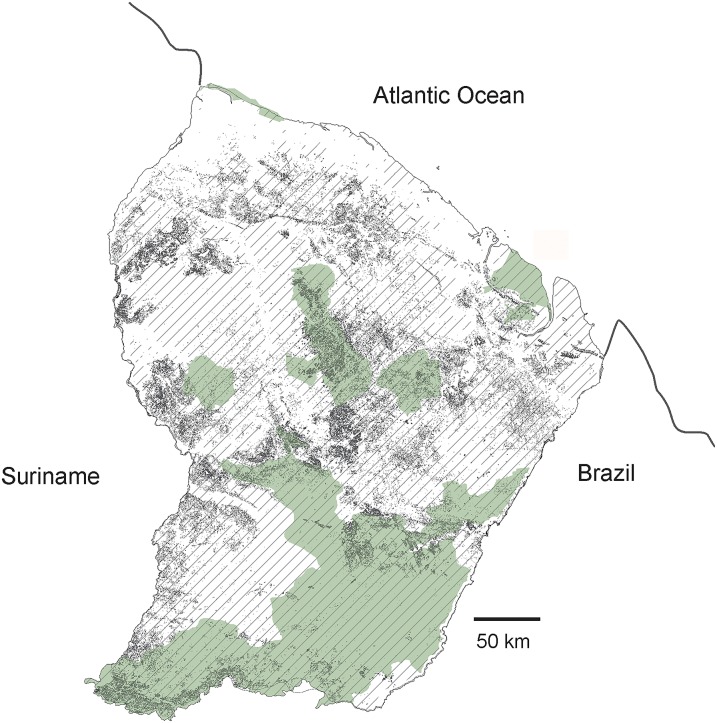
French Guiana more favorable habitats of prey. Protected areas: nature reserves and national parks, in green. Hatchings: area with Human Footprint Index < 15 (thresold value allowing maintenance of the abundance of most of prey species). Dark grey: forest areas with canopy roughness > 12, favoring higher biomass of prey.

Far more promising are the data derived from remote-sensing and radar/LiDAR analysis of forest structure. Here we showed that information based on the latest remote-sensing and plane-imaging techniques can detect fine-scale damage to the canopy cover and may inform on the status of associated fauna communities. AGB, canopy height and soil roughness informed on jaguar habitat and showed a clear relation to the predicted favorability and may highight, when surimposed with the Human Footprint index driving abundances of prey, areas of importance for long-term maintenance of ecological dynamics (Fig 5). Complex canopy structure, roughness and high AGB levels were correlated with a higher predicted occurrence of jaguar. In Chile, mesocarnivores also responded to fine-grain habitat structure attributes, identified from LiDAR, much more than to habitat, landscapes and vegetation classifications [98]. Although biotic relations between forest structure, phenology, vertebrate communities and ultimately predators will require further investigation, the high level of confidence of the relations observed between these factors already suggests that this tool will help identify key areas for conservation. Remote-sensing measurements could help detect the scale at which animals discriminate habitat characteristics, as well as the scale at which conservation priorities and plans must be implemented. Compared to categorical classifications, more gradient-based analyses of landscape architecture will yield more realistic representations of ecological heterogeneity and better predict species responses and needs [99].

Spatial and aerial images have already shown their usefulness in detecting large- and fine-scale habitat disturbance [100]. We also show here that LiDAR provides a relevant indication of forest disturbance and may easily inform on the status of habitats and associated fauna communities. Increasingly efficient tools, based on remote sensing and innovative spatial analysis methods (e.g., [101]), are being developed and will soon be operational, e.g., Sentinel 1 (radar) and Sentinel 2 (optical) satellite constellations from the European Space Agency. They will be useful to monitor tropical rainforest habitats. At this time, different techniques can be applied to obtain data dependently on the degree of precision required. In the present study, LiDAR has shown that it can estimate canopy roughness at a large scale; other techniques such as FOTO (FOurier Textural Ordination) [102] should also be explored. Furthermore, more and more open-source data are available at wide geographic scales [36]. Characterizing structure and functioning habitats to identify threats on flora and fauna are key points in the global estimation of ecosystems services in general, and of biodiversity in particular. Remote sensing provides a wide panel of investigative tools to monitor habitats and help decision makers in their conservation policies [103], and they can be particularly useful in remote forest areas as early indicators of the first signs of habitat damage and subsequent threats to animal communities.

## Supporting Information

S1 Table Sites where implemented surveys of jaguars (bold) and prey (bold and other), with projected geographic coordonates, threats, landscape categories, Human footprint, and abundance of a selected set of prey species. (XLSX)
